# 

**Published:** 1900-12

**Authors:** 


					CONTENTS.
?m?j Modern Aspects of Preventive
Medicine.
i_) A Vtt* TVT T~\
By David Samuel
Davies, M.D. Lond., D.P.H.
289
A Plea for Sanatoria for the Poorer
ponsumptives.
By Lionel A.
Weatherly, M.D.
305
Three Abdominal Cases Presenting
some Unusual Features.
W. Prichard, M.R.C.S.
313
A Case of Diffuse Leontiasis Ossea.
?Y E. H. Edwards Stack, M.B.,
F.R.C.S
316
Forty Cases of Operation for Appen-
dicitls.
By Charles A. Morton,
F.R.C.S..
320
Fiye Cases of Pelyic Disease treated
by Laparotomy.
By Ernest W.
Hey Groves, M.B., B.Sc. (Lond.)
330
The Technique of Halsted's Opera-
tion for Cancer of the Breast.
By C. Hamilton Whiteford,
M.R.C.S., L.R.C.P
3381
Progress of the Medical Sciences:
Medicine.
J. Michell Clarke,
M.D., M.A. (Cantab.)
338
Surgery.
James Swain, M.D.,
M.S. (Lond.)
343
Laryngology and Rhinology.
Barclay J. Baron, M.B.,C.M.(Ed.)
348
Otology.
W. H. Harsant
350
Reviews of Books
333
Notes on Preparations for the Sick
377
Meetings of Societies
381
The Library of the Bristol Medico-
Chirurgical Society 383
Local Medical Notes  387
Obituary
3D0
Scraps
391

				

